# Microarrays for identifying binding sites and probing structure of RNAs

**DOI:** 10.1093/nar/gku1303

**Published:** 2014-12-12

**Authors:** Ryszard Kierzek, Douglas H. Turner, Elzbieta Kierzek

**Affiliations:** 1 Institute of Bioorganic Chemistry Polish Academy of Sciences, 61-704 Poznan, Noskowskiego 12/14, Poland; 2 Department of Chemistry, University of Rochester, Rochester, NY 14627, USA

## Abstract

Oligonucleotide microarrays are widely used in various biological studies. In this review, application of oligonucleotide microarrays for identifying binding sites and probing structure of RNAs is described. Deep sequencing allows fast determination of DNA and RNA sequence. High-throughput methods for determination of secondary structures of RNAs have also been developed. Those methods, however, do not reveal binding sites for oligonucleotides. In contrast, microarrays directly determine binding sites while also providing structural insights. Microarray mapping can be used over a wide range of experimental conditions, including temperature, pH, various cations at different concentrations and the presence of other molecules. Moreover, it is possible to make universal microarrays suitable for investigations of many different RNAs, and readout of results is rapid. Thus, microarrays are used to provide insight into oligonucleotide sequences potentially able to interfere with biological function. Better understanding of structure–function relationships of RNA can be facilitated by using microarrays to find RNA regions capable to bind oligonucleotides. That information is extremely important to design optimal sequences for antisense oligonucleotides and siRNA because both bind to single-stranded regions of target RNAs.

## INTRODUCTION

Microarrays contain a library of oligonucleotides or polynucleotides with each spotted in a defined location. It is difficult to say unambiguously when microarrays appeared on the scene (1–5). Arrays with DNA attached to cellulose or another support and used for probing DNAs were precursors of currently used microarrays (6). In the 90′s, high-throughput preparation of microarrays was made possible by employment of glass as support (2) and by methods for preparation of probes, including chemical synthesis on solid support (7). Currently, microarrays are widely used to study many biological processes and to perform high-throughput analysis (8–11).

This review describes investigations in which DNA or RNA microarrays were used for identifying oligonucleotide binding sites and probing structure. Deep sequencing allows fast determination of DNA and RNA sequence. High-throughput methods for the determination of secondary structures of RNAs have been developed (12–17). These methods, however, do not reveal binding sites for oligonucleotides. In contrast, microarrays directly determine binding sites while also providing structural insights. Microarray mapping can be used over a wide range of experimental conditions, including temperature, pH, various cations at different concentrations and the presence of other molecules. Moreover, it is possible to make universal microarrays suitable for investigations of many different RNAs, and readout of results is rapid.

## EARLY-STAGE HISTORY

**Figure 1.**
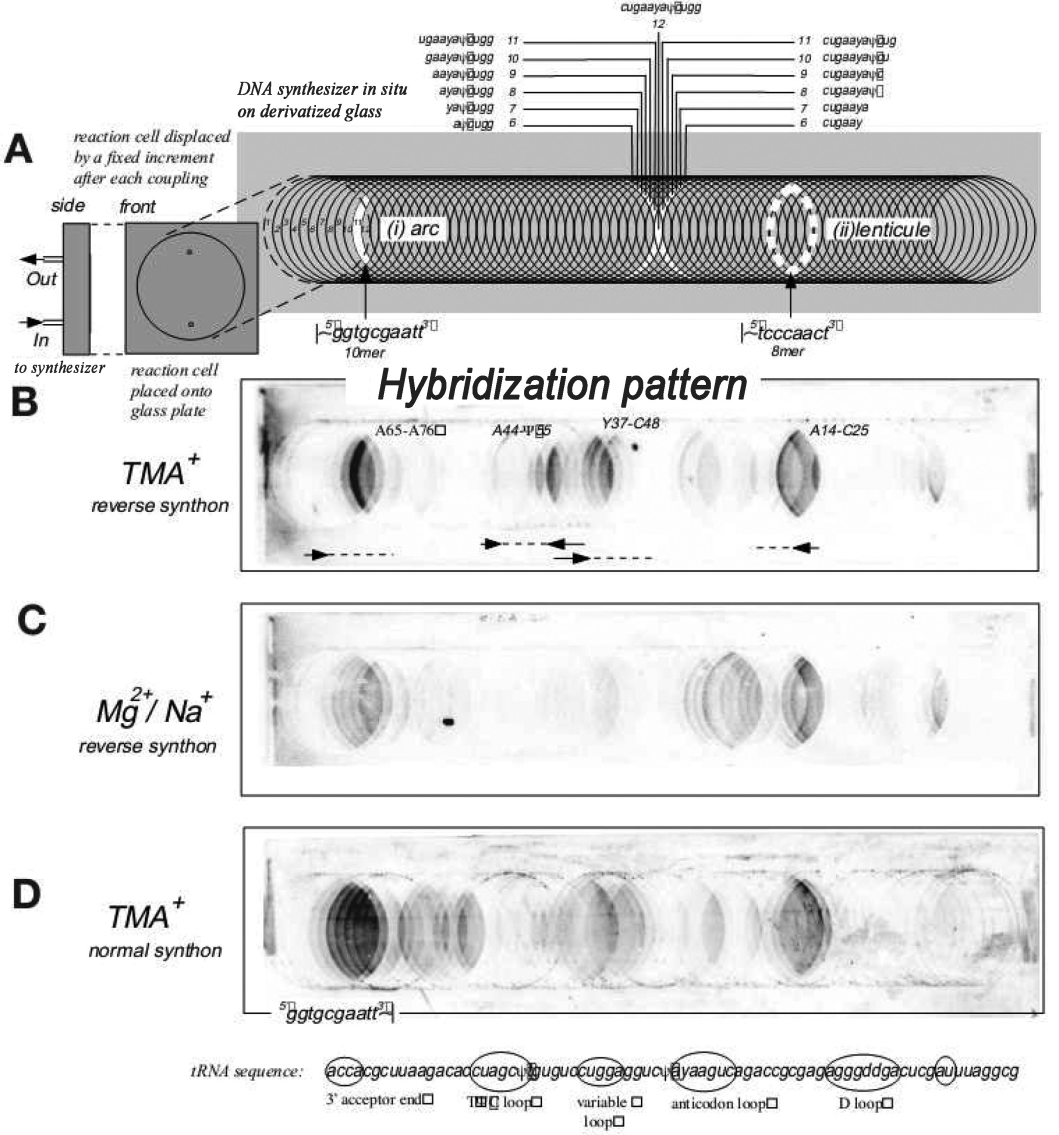
Preparation of Southern type microarrays and hybridization to tRNA^Phe^. (**A**) schematic pattern of preparation of microarray and distribution of oligonucleotide probes on it, (**B**) hybridization of target RNA in 3.5 M TMACl at 4°C to microarray in reverse synthons manner, (**C**) hybridization of target RNA in 1 M NaCl/10 mM MgCl_2_ at 4°C to microarray in a reverse synthons manner and (**D**) hybridization of target RNA in 3.5 M TMACl at 4°C to microarray in a standard synthons manner. Reprinted with permission from (21). Copyright 1999 Macmillan Publishers Ltd.

Studies of RNA structure with oligonucleotide microarrays were started by E. Southern (8,18–26). To generate DNA microarrays, DNA probes of 1–20 nucleotides were synthesized on long functionalized glass plates that were moved after each phosphoramidite coupling reaction (Figure 1) (24,27–29). Hybridization of radioactively labeled RNA to microarrays revealed binding of DNA probes to target RNA. Moreover, based on the studies of interactions of model oligonucleotides, the Southern group demonstrated that (i) probes bind to single-stranded regions of RNA and the presence of mismatches between probe and RNA sequence reduces binding intensity, (ii) strong hybridization can be observed when only a part of long probes canonically base-pairs to target RNA and (iii) the presence of secondary structural motifs adjacent to single-stranded regions influences hybridization.

The Southern group intensively studied hybridization to tRNA^Phe^ (21). Hybridization to tRNA^Phe^ was performed in the presence of 3.5 M tetramethylammonium chloride (TMACl) which reduces the sequence dependence of association constants relative to 1 M NaCl and 10 mM MgCl_2_. The results provided the following conclusions: (i) only a few of 65 dodecamer probes bind to tRNA^Phe^, (ii) the hybridization results depend on salt (3.5 M TMACl versus 1 M NaCl/10 mM MgCl_2_), (iii) only four regions of tRNA^Phe^ (acceptor stem, D stem-loop, variable loop/TΨC stem and variable loop/anticodon stem) strongly bind to the microarray probes, (iv) strength of hybridization covers two to three orders of magnitude, (v) hybridization strongly depends on RNA binding region, sometimes shifting binding region by one position results in changing hybridization yield up to 15-fold, (vi) hybridization yield is not always proportional to length of DNA probes, (vii) hybridization is the same for probes attached at the 5′ or 3′ end to the glass support. The authors observed hybridization of probes to single-stranded loops and often to adjacent helical regions. They postulated that even a short single-stranded region is sufficient for duplex nucleation and subsequent invasion of an adjacent stem, especially when the single-stranded region has A-form character and stacks on the stem. The authors concluded that ‘major determinants of hybridization lie in the structure of RNA’ (21).

The Southern group also studied application of microarrays to transcripts of 175, 336, 512, 1291 and 1389 nucleotides of the mRNA for cyclin B5 (18,19,30). Probe sequences were designed to scan only a particular fragment of 111 nucleotides. Hybridization of the transcripts to microarrays containing up to 21-nucleotide-long DNA probes showed similar patterns in certain regions of the 111 nucleotides but also different patterns characteristic for each length of transcript. Often hybridization yield of the same DNA probes was more efficient for shorter transcripts than for longer ones. They suggested that this results from more tertiary folding of longer transcripts and concluded that hybridization results can provide insight into both short- and long-range interactions. The latter can be indicated by loss of hybridization to some regions when transcript length is increased.

Results collected by the Southern group were mostly used to select antisense oligonucleotides to achieve efficient gene silencing. For example, they studied three *Xenopus laevis* B1, B4 and B5 cyclins mRNAs ranging in size between 420 and 1400 nucleotides (19). They scanned abilities of 21-nucleotide-long DNA probes to hybridize to the first 120 nucleotides of those mRNAs. Often probes bound to specific regions in all three mRNAs, but strong hybridization was not correlated with probe base composition, presumably because of secondary structure in the target RNA. Predicted secondary structures of target mRNAs indicated that probes often bind intensively to helical regions. The microarray results provided optimal antisense oligonucleotides to the first 120-nt region. Selected antisense oligonucleotides were then assayed for their effect on translation of endogenous cyclin mRNAs in *Xenopus* egg extracts and their ability to promote RNase H cleavage of cyclin mRNAs in oocytes. DNA oligonucleotides identified as strong binders in microarray experiments inhibited translation of their cognate targets in *Xenopus* egg extract and strongly reduced synthesis of the targeted cyclins, whereas those oligonucleotides with weak binding to microarrays produced no or little inhibition. Generally, the authors demonstrated the relationship of target site and oligonucleotide sequence with antisense activity. The authors suggested that many factors could be responsible for behavior, including sequence composition and length of DNA probes, secondary structures of target RNAs and less favorable thermodynamic stability of DNA/RNA duplexes over intramolecular RNA/RNA helixes.

## DEVELOPING RNA-LIKE AND ISOENERGETIC MICROARRAYS FOR PROBING BINDING

The Kierzek and Turner groups developed a new approach to study RNAs with oligonucleotide microarrays (31–37). In this approach: (i) the probes are 5- or 6-nucleotides long and based on the natural 2′-O-methylated backbone because single-stranded regions within natural RNAs are short and 2′-O-methylRNA/RNA duplexes are more stable (38) than RNA/RNA (39) or DNA/RNA (40) duplexes, and (ii) the probes are modified to make hybridization to unstructured RNA relatively independent of base sequence, i.e. probes are isoenergetic.

Short isoenergetic probes simplify the interpretation of probe binding, which depends on differences between the free energies of breaking self-structure of probe and target RNA and formation of duplex between probe and target. Penta/hexanucleotide probes can be assumed to have no self-structure. Moreover, short probes facilitate differentiating between strong binding complementary and weaker binding mismatched probes by changing hybridization temperature. Due to smaller enthalpy change for short duplexes, the difference in melting temperature (Tm) between complementary and mismatched probes is larger than for long probes.

### Standard microarrays and structural studies of 5S rRNA–short oligonucleotides can give sufficient binding

Initial experiments were performed on microarrays with 2′-O-methylated (2′OMe) heptanucleotide probes (37). Ribosomal 5S RNA from *Escherichia coli* was selected as target because computer programs such as Mfold (41) and RNAstructure (42) predict only 27% of the known canonical base pairs of the native structure. Hybridizations of ^32^P-labeled 5S rRNA were performed in various conditions and results used as constraints in folding of target RNA with the RNAstructure program. In particular, the 5S rRNA nucleotide complementary to the middle nucleotide of a strongly binding heptanucleotide probe was constrained not to be canonically base-paired. The best hybridization results were obtained in buffer containing 1 M NaCl, 4 mM MgCl_2_ and 10 mM Tris-HCl, pH 7.4 at room temperature (RT) and at 4°C, where probe binding was significantly enhanced relative to 0.15 M NaCl, 4 mM MgCl_2_ and 0.04 M NaCl, 10 mM MgCl_2_. Strong hybridization was reported to loops B and C but not loop E, which is known to be unusually stabilized by Mg^+2^ (43). Thus, microarrays can distinguish between loops with strong and weak local structure.

In the absence of Mg^+2^, *E. coli* 5S rRNA forms a different secondary structure (44,45). When 5S rRNA was mapped with chemicals and microarrays at 1 M NaCl, the patterns differed dramatically from those observed in the presence of Mg^+2^ (37).

The results with 5S rRNA revealed advantages and disadvantages of the microarray approach. Advantages of short probes include limited interference with target RNA secondary structure and ability to have probes representing all possible sequences. Another advantage is that probe binding often does not completely overlap with chemical modification, so that complementary information is generated. A disadvantage is that a short probe may have more than one binding site. This is particularly problematic because A-U and G-U pairs have similar stability (46–48). Replacing uridine with 2-thiouridine (s^2^U), however, improves discrimination between A-U and G-U because A-s^2^U is more stable than A-U, whereas G-s^2^U is less stable than G-U (37,49,50). Another disadvantage is that the binding constant for unmodified probes is very sequence dependent, which complicates interpretation. Moreover, binding constants are low for probes with high A/U content. For example, thermodynamic stabilities (free energy, ΔG^o^_37_) of 5′(AmUm)_3_/3′(UA)_3_ and 5′(GmCm)_3_/3′(CG)_3_ duplexes are −2.8 and −12.2 kcal/mol, respectively, whereas melting temperatures (in 1 M NaCl) are 11 and 71°C, respectively (calculated based on (51), (http://rnachemlab.ibch.poznan.pl/calculator2.php)). This disadvantage can be eliminated, however, by using modified probes as described below.

### Isoenergetic microarrays – modifications provide roughly sequence-independent binding to simplify interpretation

Interpretation of binding is greatly simplified if all probes have the same binding constant to an unstructured RNA. One approach to equalizing binding constants to unstructured RNA is to replace some 2′-O-methylated nucleotides with locked nucleic acid (LNA) residues (51–53). Thermodynamic studies found that isolated LNA residues at internal positions enhance stabilities (ΔΔG^o^_37_) of LNA-2′OMeRNA/RNA duplexes by ca. 1.5 ± 0.5 kcal/mol per single LNA nucleotide, which increases binding constant by roughly 10-fold at 37°C. Little or no additional substitution is achieved if an adjacent LNA is added. This phenomenon may be caused by *C3*′*-endo* conformation of LNA nucleotides forcing 3′-adjacent nucleotide also to adopt *C3*′*-endo* conformation and thus facilitate formation of A-form helix (54). At terminal positions the contribution of LNA residues on stabilities is ca. 0.3–1.4-kcal/mol/LNA nucleotide. Internal LNA–RNA mismatches destabilize LNA-2′OMeRNA/RNA duplexes between 1.5 and 7 kcal/mol depending on type of mismatch and position within duplex. Destabilizations are less at terminal positions. The results revealed that it is optimal to place LNA residues at every second or even third nucleotide within a strand and to avoid placing LNA residues at terminal positions. As described below, an exception to the latter rule is that a terminal LNA G is often added as an extra nucleotide to increase stability.

A second approach to improve thermodynamic stabilities is to replace A with 2,6-diaminopurine riboside as either a 2′-O-methyl or LNA nucleotide (55). The 2,6-diaminopurine residue interacts with uridine via three hydrogen bonds and improves stability by ca. 1 kcal/mol relative to A.

**Figure 2.**
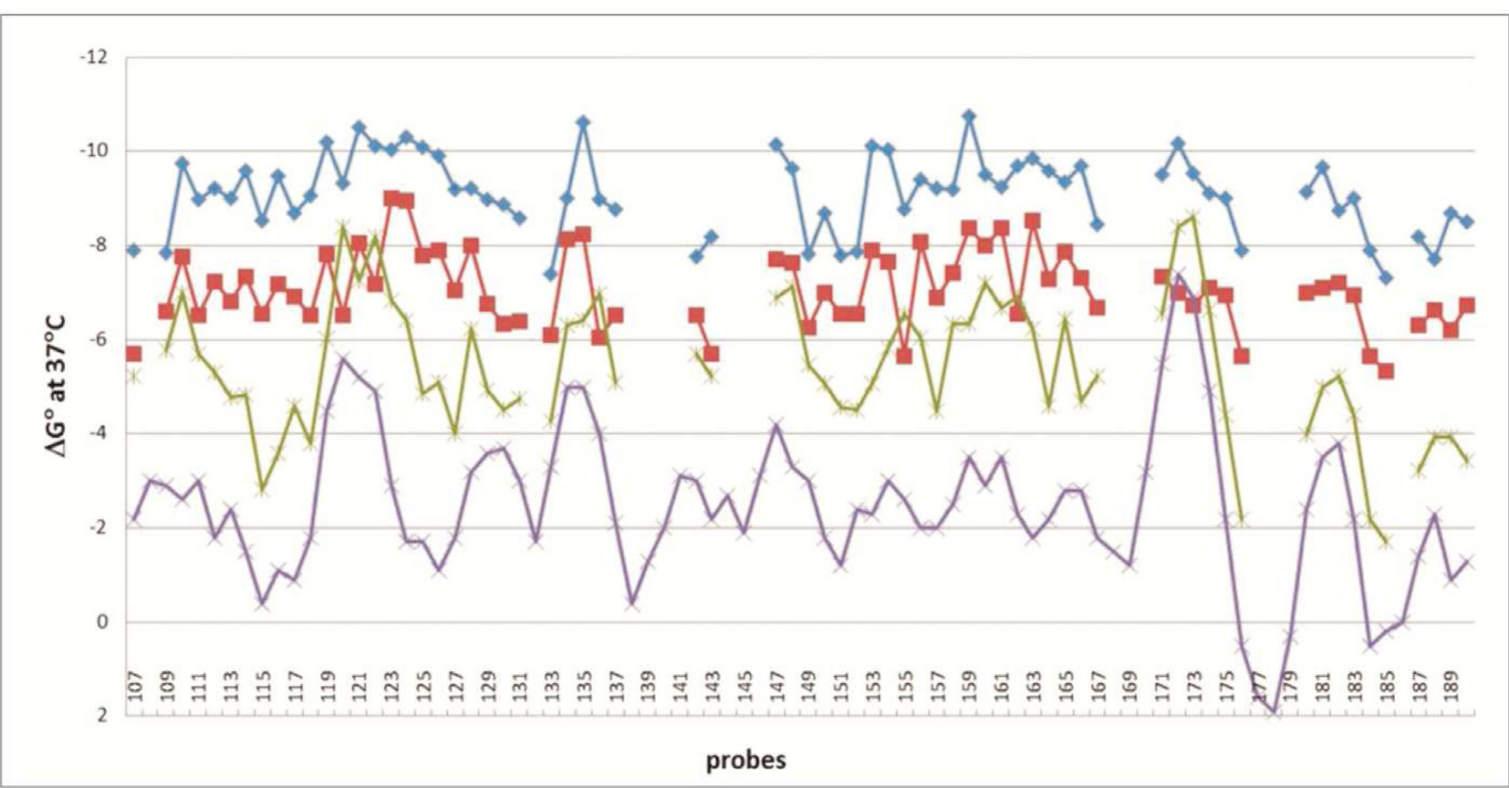
Comparison of calculated free energies of duplexes formed by DNA (purple line), 2′-O-methylRNA (green line), 2′-O-methylRNA including 2′-O-methyl-2,6-diaminopurine riboside (red line), isoenergetic probes (LNA and 2′-O-methylRNA including 2′-O-methyl-2,6-diaminopurine riboside) (blue line) and complementary single-stranded sequence fragments of RNase P RNA from *Bacillus subtilis* (RNRspBs). This plot has corrections to the plot on Figure S2 of (62).

Based on many 2′OMeRNA/RNA microarray studies, the free energy change at 37°C (ΔG^o^_37_) has to be more favorable than −6 kcal/mol to observe clearly detectable hybridization signals. For hybridization to target sequences with high A/U content it is often difficult to get ΔG^o^_37_ more favorable than −6 kcal/mol. For this reason, a third approach for designing isoenergetic probes is to add LNA-G to the 3′-end. Model studies showed that 3′-terminal mismatches G^L^-A, G^L^-U and G^L^-G stabilize 2′OMeRNA/RNA duplexes by 1.6, 1.5 and 1.4 kcal/mol, respectively, at 37°C (56). Formation of 3′-terminal G^L^-C pairs, however, stabilizes the same model LNA-2′OMeRNA/RNA duplex by 3.4 kcal/mol, which adds ambiguity to the identification of binding site. Figure 2 shows an example of differences made when probes are designed to be strongly binding and isoenergetic. When unmodified DNA probes are used, ΔG^o^_37_ for binding to unstructured RNA is predicted to oscillate around −3.5 ± 2.5 kcal/mol. In contrast, 2′OMeRNA probes modified with LNA and 2,6-diaminopurine riboside give binding predicted to oscillate around −9 ± 1 kcal/mol (Figure 2).

There are 1024 different pentamer sequences. On the basis of thermodynamic predictions, it was possible to synthesize around 850 penta/hexanucleotide LNA-2′OMeRNA/RNA relatively isoenergetic probes with ΔG^o^_37_ values favorable enough for use in microarrays. Of the 850 probes, around half were with 3′-terminal LNA G. Probes contained a 5′-end aminohexyl linker allowing them to covalently bind to oxidized agarose-coated microscope slides (57,58). Oligonucleotide spots with diameters of ca. 0.15 mm and separated by 0.75 mm are automatically printed on a functionalized microscope slide. Thus, ∼2000 probes can be spotted on a single microarray. Usually spots are printed in triplicate, including several controls and reference probes. Therefore, two microarrays can be designed to probe essentially any RNA.

#### Studies of retrotransposon R2 5′RNAs–large RNAs can be studied

**Figure 3.**
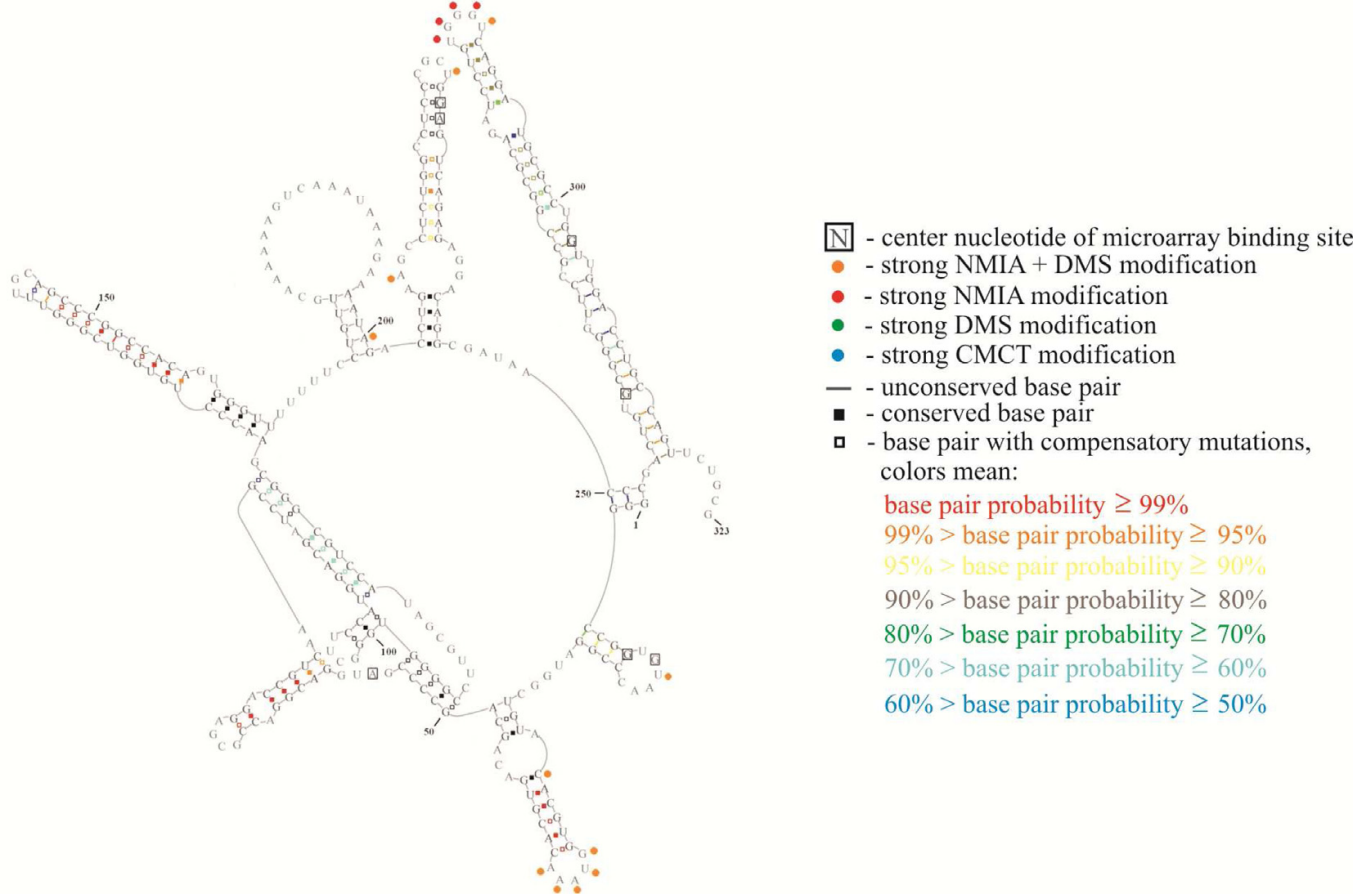
Structural model for *Bombyx mori* 5′ RNA (R2Bm). Structure is annotated with modeling constraints generated from strong and medium binding to oligonucleotide microarrays (boxed nucleotides) and from chemical mapping (green, blue, red and orange circles, respectively, corresponding to strong reactivity with DMS, CMCT, NMIA and CMCT or DMS overlapping with NMIA). Also annotated are base-pair conservation (open boxes between nucleotides), compensatory mutations (filled boxes between nucleotides) and partition function probabilities (colored boxes or dashes between nucleotides) (34).

The first structural studies using isoenergetic microarrays were performed on the roughly 330-nt 5′-regions of R2 retrotransposon RNA from *Bombyx mori* (R2Bm), *Samia cynthia* (R2Sc), *Coscinocera hercules* (R2Ch), *Callosamia promethean* (R2Cpr) and *Saturnia pyri* (R2Spy) (Figure 3) (34,36). Initially, only the *B. mori* sequence was available and the secondary structure was unknown. Hybridizations were performed in the presence of 5 mM MgCl_2_ with 0.2 M or 1 M of NaCl and at 4°C or RT. Optimal results were obtained at 0.2 M NaCl, 5 mM MgCl_2_, 10 mM Tris-HCl, pH 8, RT. In principle, mapping an RNA containing ‘n’ nucleotides with shifting of probes by one position requires n-4 probes. In reality, the number of probes is lower because only probes with free energies of hybridization more favorable than −6 kcal/mol are synthesized. For example, studies of R2Bm that contains 323 nucleotides used 232 instead of 319 probes. The average predicted free energy (ΔG^o^_37_) of binding of the modified probe library to complementary sites of the R2Bm 5′ RNA if the sites were single stranded was −9.8 ± 1.2 kcal/mol.

Based on microarray mapping, a secondary structure of R2Bm 5′ RNA was predicted (36). After omitting 27 probes with alternative binding sites, only seven probes with unambiguous binding sites were used as constraints. The same target RNA was chemically mapped with dimethyl sulfate (DMS), 1-cyclohexyl-(2-morpholinoethyl)carbodiimide metho-p-toluene sulfonate (CMCT), 1,1-Dihydroxy-3-ethoxy-2-butanone (kethoxal) and *N*-methylisatoic anhydride (NMIA). The same RNA secondary structure was predicted by RNAstructure from thermodynamics with microarray constraints alone as with constraints from both microarray and chemical mapping data (36). Interestingly, fragment 50–123 of R2Bm 5′RNA was not hit strongly by chemical reagents even though there were many nucleotides predicted to be single stranded. This suggested alternative folding to a pseudoknot. At the time, pseudoknots were not allowed in algorithms predicting secondary structure on the basis of thermodynamics and experimental constraints. Nuclear magnetic resonance spectra, however, proved the pseudoknot folding (59). The pseudoknot and most of the rest of the secondary structure was confirmed by sequence comparison when four additional sequences became available (34). One test of a structure is whether it has reasonable binding sites for strongly binding probes not used as constraints because there are more than one potential binding site. With exception of probes 227 and 228, all 26 probes with multiple potential binding sites have at least one site in the proposed R2Bm 5′RNA secondary structure that would obviously be a good binding site. Probes 227 and 228 would be expected to bind if the top of helix P7b can shift up or down by one nucleotide to form an internal C-A pair. Equivalent studies of the other four R2 5′RNAs revealed conserved structures of hairpins P1, P6, P7 and P8, and the pseudoknot containing P2, P3, P4 and P5.

Binding of oligonucleotide probes identifies regions of weak or no intramolecular base pairing in RNA. Interestingly, of 14 probes with unambiguous or very probable binding sites in R2Bm 5′RNA, only one of the central target nucleotides is strongly modified during chemical mapping. It demonstrates again that microarray mapping can provide information complementary to that available from chemical mapping.

#### Studies of models for RNA structural motifs—invasion of terminal helixes

Studies of model systems with hairpins, internal loops, bulges, 3′- and 5′-dangling ends and pseudoknots revealed some factors that influence binding (32). The models had short helixes because natural RNA helixes are typically short, containing ca. 6–8 base pairs. All model RNAs were UV-melted to determine thermodynamic stability to assure that each model RNA folded exclusively into one defined structure under chosen hybridization conditions. The models were hybridized to isoenergetic microarrays in various conditions, including different concentrations of NaCl, MgCl_2_ and temperature.

**Figure 4.**
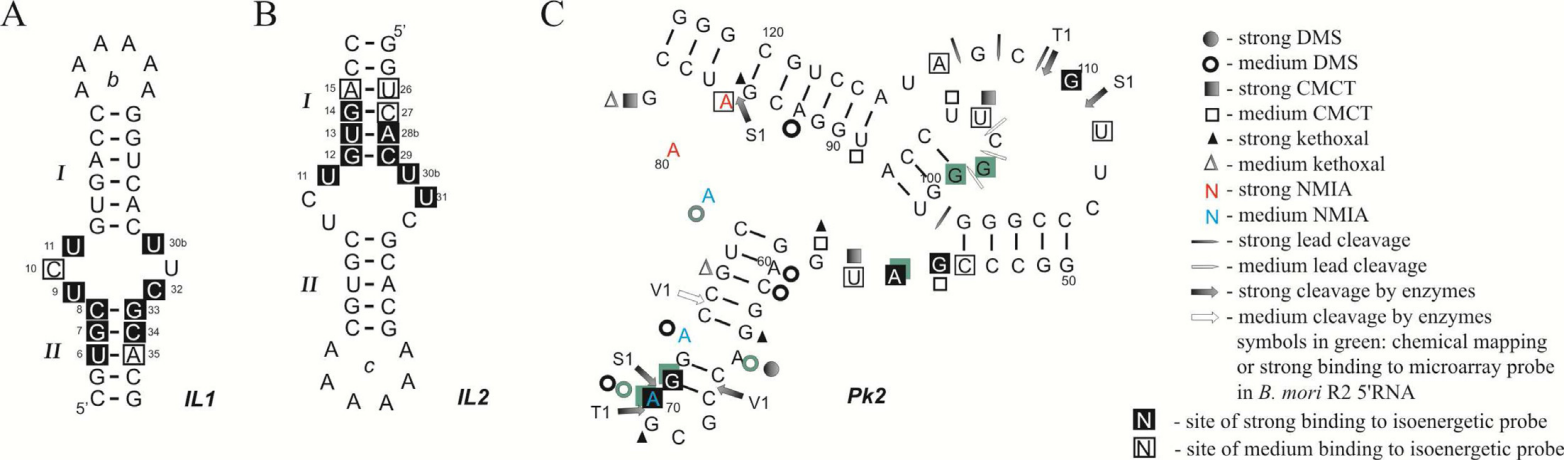
Binding of isoenergetic oligonucleotide probes to model hairpins and pseudoknot (32). In hairpin IL1 (**A**) loop b terminates helix I, whereas in hairpin IL2 (**B**) loop c closes helix II. Inversion of the element terminating a helix correlates with inverting binding ability to the adjacent helical region. (**C**) Secondary structure and hybridization results for 74-pseudoknot (Pk2), a part of *B. mori* R2 5′RNA. Hybridization was performed in 200 mM NaCl, 5 mM MgCl_2_ and 10 mM Tris-HCl, pH 8.0 at room temperature. Pk2 was hybridized to a microarray with all possible isoenergetic oligonucleotide probes complementary to Pk2. Filled in squares indicate site of strong binding to isoenergetic probe (middle nucleotide of region binding to pentamer probe), open squares indicate site of medium binding to isoenergetic probe. See the legend for meaning of other symbols.

Binding to hairpin loops was observed, as expected. However, binding to terminal helixes was also observed often, usually by extension of binding to nucleotides not canonically paired (Figure 4A and B). Interestingly, helix invasion was more likely from a 5′ than a 3′ trinucleotide dangling end. This trend could reflect the larger helix stabilization provided by a 3′-dangling end relative to a 5′-dangling end. For example, a 5′-unpaired UCU is predicted to enhance free energies of a helix (ΔG_37_^o^) by 0.1–0.4 kcal/mol at 37°C, whereas a 3′-unpaired UUC stabilized by 1.0–1.9 kcal/mol (60,61). Two pseudoknots were also studied, one of the 74 nucleotides from the *B. mori* R2 retrotransposon (Figure 4C) and a second of 32 nucleotides composed of two RNA hairpins (32,34). In both cases probe binding was observed to single-stranded regions. Surprisingly, the isolated R2 pseudoknot bound 11 probes (Figure 4C) compared to only one in the 323 fragment (Figure 3). It was also much more reactive to chemicals.

The analysis of RNA structural motifs binding to microarray probes demonstrates the following features: (i) bulges, internal loops and dangling ends bind strongly to microarray probes; (ii) a terminal stem can also bind strongly, presumably due to invasion of stem by the modified oligonucleotide; (iii) internal stems do not typically bind strongly. Along with further results described below, the data demonstrate that the location of the structural motif in the context of the whole target structure and both secondary and tertiary interactions influence the accessibilities of RNA to probe binding (32,33,62,63).

#### Studies of RNase P RNA—binding to an internal helix but not to a large internal loop

**Figure 5.**
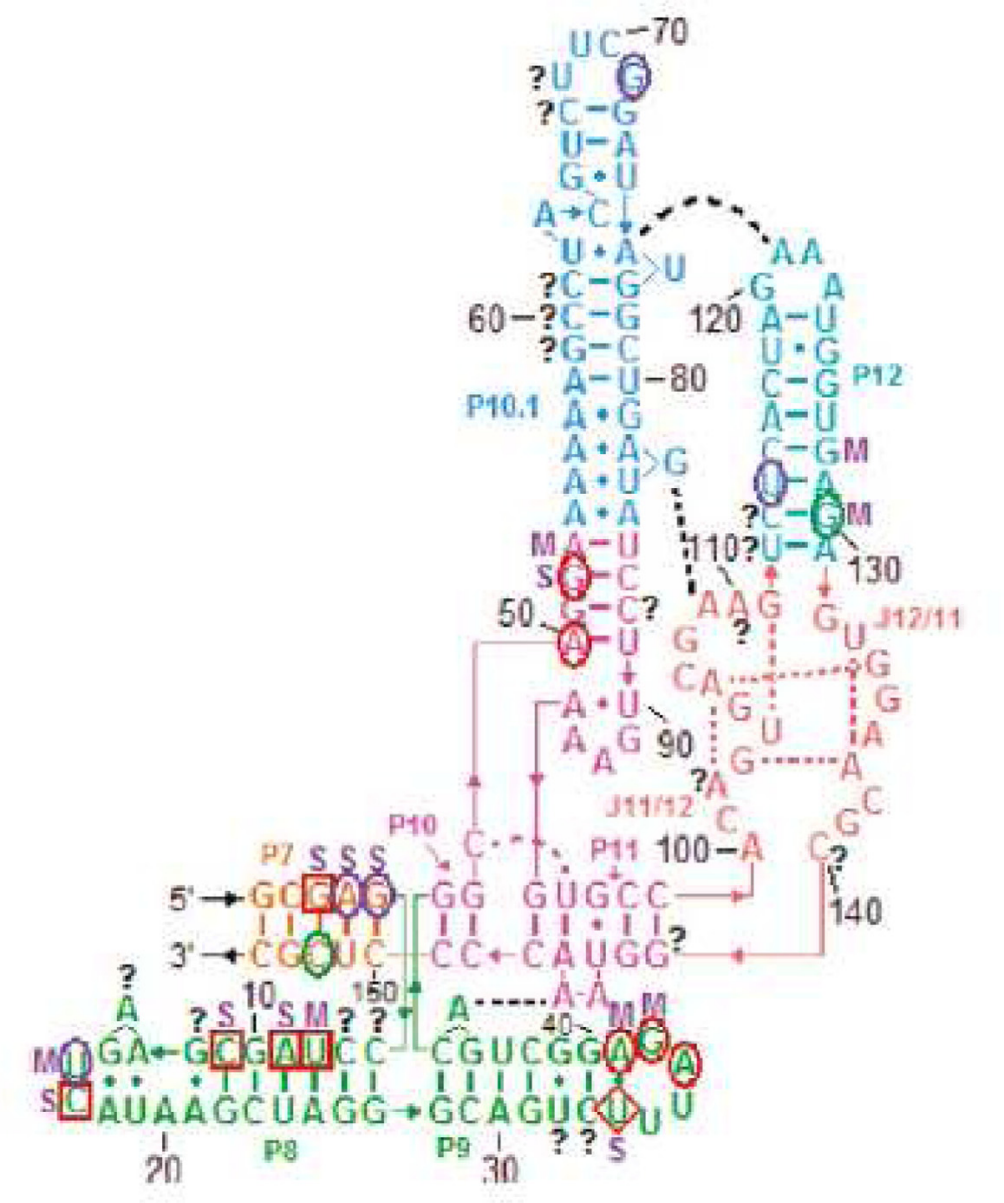
Binding sites of RNRspBs by isoenergetic probes on microarrays explored in three buffer conditions. Red squares and circles, respectively, indicate strong and medium binding in 140 mM NaCl, 80 mM HEPES, 10 mM MgCl_2_ and also 135 mM KCl, 25 mM NaCl, 50 mM HEPES, 10 mM MgCl_2_. Purple circles indicate medium binding only in 135 mM KCl, 25 mM NaCl, 50 mM HEPES, 10 mM MgCl_2_. The red diamond indicates medium binding in 140 mM NaCl, 80 mM HEPES, 10 mM MgCl_2_ and strong binding in 135 mM KCl, 25 mM NaCl, 50 mM HEPES, 10 mM MgCl_2_. Green circles indicate medium binding in 140 mM NaCl, 80 mM HEPES, 10 mM MgCl_2_. S indicates strong binding in 135 mM KCl, 25 mM NaHEPES, 50 mM HEPES, 1 M NaCl. M indicates medium binding in 135 mM KCl, 25 mM NaHEPES, 50 mM HEPES, 1 M NaCl. Question marks indicate ambiguous binding sites. Reprinted with modifications and permission from (64). Copyright 1999 Macmillan Publishers Ltd.

Microarray studies (62) on the 154-nucleotide construct of the specificity domain of RNase P RNA from *Bacillus subtilis* (RNRspBs) that was used to solve the crystal structure (64) revealed unexpected probe binding (Figure 5). While three of the four hairpin loops at most bind probes as expected, a terminal (P7) and an internal (P8) helix also bind strongly. Most surprisingly, a large internal loop at most possibly bound only two ambiguous probes. This RNA has a complex crystal structure and chemical mapping in solution is consistent with this structure. There are several potential reasons for the unexpected binding, including oligonucleotide trapping of minor species (i.e. structural rearrangement (65)) and helix invasion stabilized by probe modifications and/or triple helix formation.

Many approaches are being developed to target RNA with oligonucleotide mimics as therapeutics. It would be advantageous to develop approaches that use short oligonucleotides. The results for RNRspBs show that even a crystal structure does not allow obvious design of suitable oligonucleotides. Microarray experiments, however, rapidly reveal binding sites in target RNAs. The results suggest also that short oligonucleotides may induce refolding of RNA into an alternative conformation that could abrogate function.

#### Studies of influenza RNA—identifying potential sites for chemical genetics and therapeutics

Isoenergetic microarrays are being used to probe sequences in influenza A that are predicted to fold into stable, conserved structures (66–69). In the presence of Mg^+2^, the 3′-splice site of influenza A segment 7 mRNA is in equilibrium between a hairpin and a pseudoknot conformation. In the two conformations, the splice site and other functional elements exist in different structural environments, suggesting that splicing can be controlled by a conformational switch that exposes or hides the splice site similarly as postulated for HIV-1 RNA (65,70). Only the hairpin is present in the absence of Mg^+2^ and only the pseudoknot is present when Mg^+2^ is replaced with Co(NH_3_)_6_^+3^. Microarray probing of the two conformations revealed different patterns of binding, suggesting that splicing could be regulated by oligonucleotides. Because both proteins encoded in segment 7 are essential, the splice site is a potential therapeutic target.

The functions of two other structures predicted by bioinformatics (71) are unknown. Microarray results identify sites of oligonucleotide binding, suggesting that a ‘chemical genetics’ approach with modified oligonucleotides could help reveal the function of these structures.

#### Model studies of tRNAs—small molecules but a lot of information

Microarray mapping of tRNAs revealed the ability of microarrays to detect positions of modifications that affect base pairing and also subtle differences in local structure.

**Figure 6.**
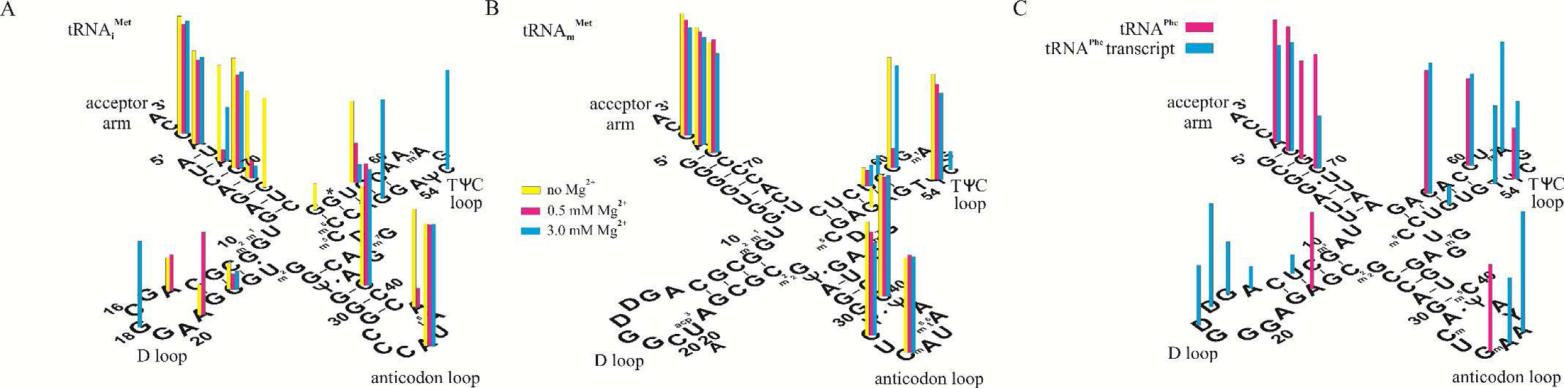
Isoenergetic microarray mapping results for tRNA_i_^Met^ (**A**) and tRNA_m_^Met^ (**B**) in 1 M NaCl, 20 mM sodium cacodylate, 0.5 mM Na_2_EDTA pH 7.5 (yellow bars), 1 M NaCl, 0.5 mM MgCl_2_, 10 mM Tris-HCl pH 7.5 (red bars) and 1 M NaCl, 3.0 mM MgCl_2_, 10 mM Tris-HCl pH 7.5 (blue bars). The height of the bar corresponds to strength of probe binding. Isoenergetic microarray mapping results for tRNA^Phe^ (**C**) (red bars) and unmodified transcript (blue bars) in 1 M NaCl, 0.5 mM MgCl_2_, 10 mM Tris-HCl, pH 7.5 at 4°C. The height of the bar corresponds to strength of probe binding (33,63).

In nature, only initiator tRNA_i_^Met^ can denote the first amino acid in polypeptide synthesis and only the elongator tRNA_m_^Met^ allows extension of polypeptide. Studies on initiator tRNA_i_^Met^ and elongator tRNA_m_^Met^ from *Lupinus luteus* showed that differences in structure of those tRNAs produce different patterns of probe binding (Figure 6A and B) (33). One difference is accessibility of the D loop in tRNA_i_^Met^ and lack of binding to the D loop of tRNA_m_^Met^. This reflects the lack of poorly pairing dihydroU nucleotides in the D loop of tRNA_i_^Met^.

A second difference concerns binding to the anticodon loop. The binding sites of tRNA_i_^Met^ are 35 and 38, whereas in tRNA_m_^Met^ binding sites are 32 and 34. Generally, the 5′-side of the anticodon loop of tRNA_m_^Met^ is accessible for binding, whereas in tRNA_i_^Met^ only the central and 3′-side are accessible. Structural changes induced by MgCl_2_ also change microarray binding. For example, high concentration of NaCl (1 M) and 3 mM MgCl_2_ in hybridization buffer is required for binding at sites 52 and 56 of the tRNA_i_^Met^ TΨC loop. The tRNA_m_^Met^ TΨC loop is more accessible for probes.

Studies on *Saccharomyces cerevisiae* tRNA^Phe^ and its unmodified RNA transcript provide another demonstration of microarrays revealing positions of modifications (Figure 6C) (63). Differences in binding were found in each hairpin loop, all of which are modified in natural tRNA^Phe^. Generally, fewer binding sites and weaker binding were observed in natural tRNA^Phe^ than its unmodified transcript. RNA modifications such as dihydrouridine (D), N^2^-methylguanosine (m^2^G) and N^2^,N^2^-dimethylguanosine (m^2^_2_G) in internal positions influenced thermodynamic stabilities of hybridization duplexes (72,73). Not every change in probe binding, however, can be explained by reduced duplex stability. Part of the differences could originate from tertiary interactions between D and TΨC loops in tRNA^Phe^ and different interactions in transcript tRNA^Phe^. Comparison of tRNA^Phe^ mapping with chemical and enzymatic methods provides similar structural conclusions (74–76).

### Studies of RNA within binary and tertiary complexes

**Figure 7.**
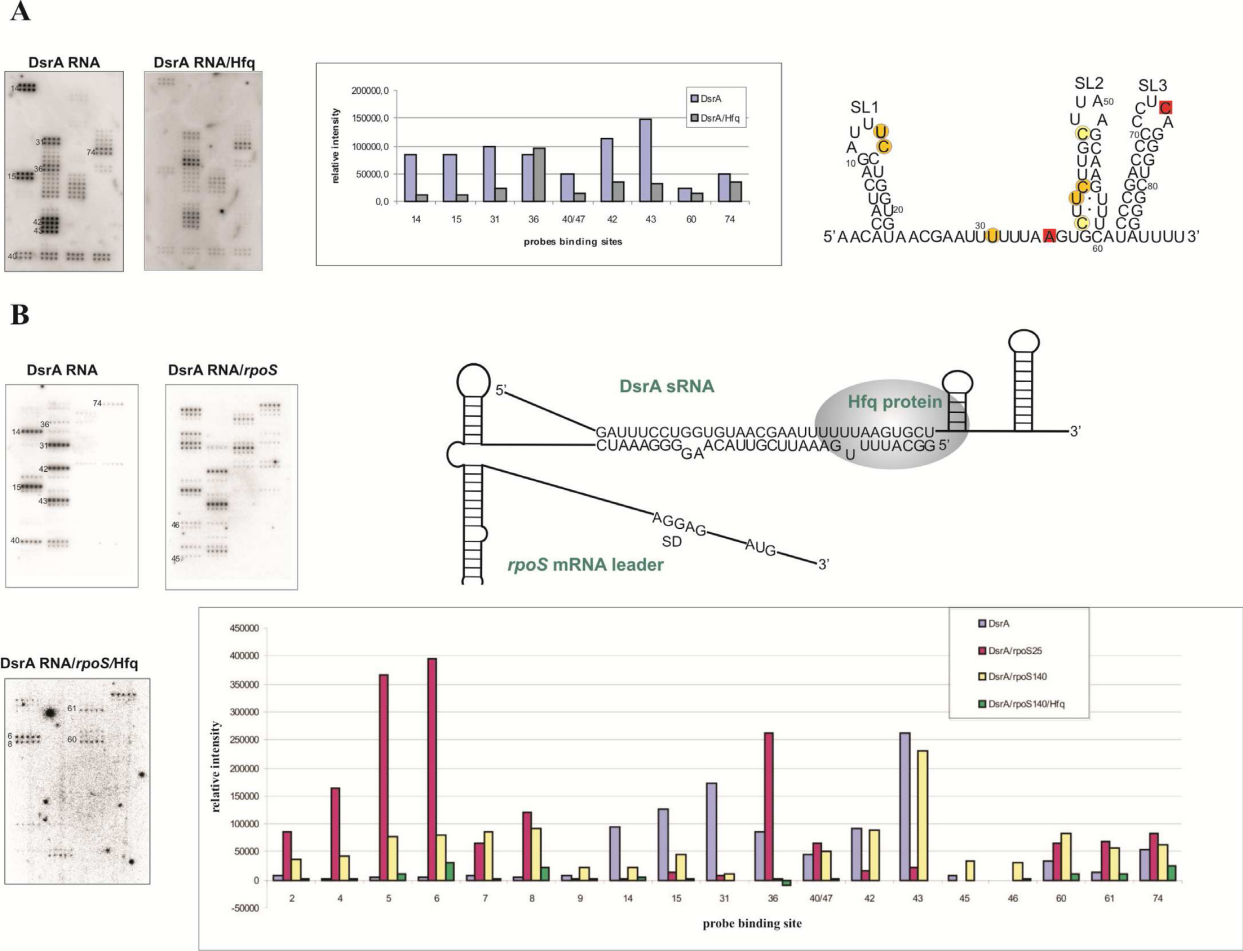
(**A**) Isoenergetic microarrays mapping results for DsrA sRNA in complex with Hfq. On the left side, microarrays with hybridized DsrA RNA and complex of DsrA RNA/Hfq. On the right side, the bars are related to intensity of binding to probes (at selected positions) of DsrA RNA in the absence and presence of Hfq. On secondary structure of DsrA RNA red squares represent sites of binding that have the same intensity in the absence and presence of Hfq. (**B**) On the left side, mapping with isoenergetic microarrays DsrA RNA alone, complex DsrA RNA/*rpoS* mRNA and complex DsrA RNA/*rpoS* mRNA/Hfq. On the right side, schematic presentation of the interactions of DsrA RNA*, rpoS* mRNA and Hfq protein. The bar graph demonstrates intensity of binding to probes (at selected positions) of DsrA RNA in the absence and presence of Hfq. Various patterns hybridization of DsrA RNA is related with different positioning probes (77).

Microarray mapping can also be used to study RNA in complex with protein and/or other RNAs (77). The first models for such studies were non-coding DsrA RNA and OxyS RNA bound to Hfq protein. In principle, only single-stranded fragments of RNAs not involved in any type of interactions will bind to probes (Figure 7). Formation of complexes can thus change probe binding patterns in a microarray.

Formation and stabilities of complexes were first analyzed by gel electrophoresis. Then hybridizations were performed with DsrA alone and with Hfq. The microarray mapping of isolated DsrA confirmed its published secondary structure (78,79). As shown in Figure 7, microarray mapping in the presence of protein reduced microarray binding to sequences expected to bind protein. Similar experiments with 25- and 140-nt fragments of rpoS mRNA (rpoS25 and rpoS140) and Hfq revealed an Hfq-induced change in rpoS secondary structure (Figure 7) (77).

Inside cells, DsrA RNA regulates translation of many mRNAs, including *rpoS* mRNA. It was postulated that the regulation is achieved by interactions of DsrA RNA and the 5′UTR of *rpoS* mRNA (80). Hybridization of the rpoS140/DsrA RNA complex to probes on isoenergetic microarrays revealed binding sites and demonstrated that the interaction region of DsrA RNA and *rpoS* mRNA is longer than previously reported. Microarray studies of the DsrA RNA/Hfq/rpoS 140 RNA revealed that some probes no longer bind and that the main Hfq binding site is probably displaced to another site of DsrA RNA and that Hfq interacts with *rpoS* mRNA outside the region of complementarity to DsrA RNA.

### Other approaches to generating isoenergetic microarrays

#### Isostable DNA microarrays

The Richert group in 2007 developed DNA modifications that could be used to make ‘isostable DNA’ microarrays (81). To make ‘isostable DNA’, they introduced pyrene or anthraquinone derivatives into DNA probes and in some cases trimethoxystilbene at the 5′-terminus. Moreover, LNA, particularly LNA-thymidine and LNA-adenosine were incorporated at terminal positions. To reduce thermodynamic stability of G-C pairs, 2′-deoxyguanosine or 2′-deoxycytidine was replaced with 2′-deoxyinosine or 2′-deoxy-N4-ethylcytidine, respectively. Presumably, these DNA microarrays could also be used to probe RNA, although different design rules would be necessary because the sequence dependence of DNA/RNA and DNA/DNA duplexes differs (40,82). Moreover, DNA probes are less likely to maintain RNA conformations.

#### Unmodified 2′-O-methylated microarrays

The Verdine group (83) used microarrays fabricated by photoresist lithography and consisting of all possible unmodified 2′-O-methylated tetramers to octamers. They tested their method on the 451-nucleotide fluorescently labeled human telomerase RNA (hTR) and on shorter constructs including two pseudoknots. Consistent with previous studies (21,22,24,37,62), probes that bound to single-stranded regions could invade adjacent helixes, especially those formed between the 5′ and 3′ ends of an RNA.

## SUMMARY OF LESSONS FROM PUBLISHED STUDIES

Isoenergetic oligonucleotide microarray mapping is based on interactions of oligonucleotide probes with target RNAs. Hybridizations can be performed over a wide range of buffer, cations, temperature, time and pH conditions. This contrasts with enzymatic mapping that requires conditions favorable for enzyme activity, e.g. the presence of specific divalent cations and limited pH. The same concerns chemical mapping since to react with target RNA the chemical reagents (DMS, CMCT, kethoxal, NMIA, 1M7) require conditions that could influence the structure of RNA. For example, some reagents used for chemical mapping require presence in reaction mixture of ethanol (for CMCT and kethoxal) or dimethylsulfoxide (for NMIA).

Moreover, microarray mapping is simple and fast. The 850 penta/hexanucleotide probes that form hybridization duplexes thermodynamically stable enough for mapping can be multiply printed on a large number of single microscope slides that can then be used to map any RNA.

Of course, isoenergetic microarrays have limitations. Pentanucleotide probes can have multiple complementary binding sites, particularly in large RNAs. Moreover, U binds to A and G with roughly equal stability (47). Additionally, probes can occasionally bind to canonically paired helixes, especially those formed by the 5′ and 3′ ends of the RNA (37,62,83). All those effects complicate interpretation. Based on published experiments, microarray mapping is particularly useful for RNAs containing up to 300–500 nucleotides. Unfortunately, isoenergetic microarrays are not yet commercially available.

## FUTURE IMPROVEMENTS AND POTENTIAL

Isoenergetic oligonucleotide microarrays can now quickly identify regions accessible to bind oligonucleotides, which are therefore potential targets for therapeutics or chemical genetics experiments. In concert with other methods, they can also reveal regions protected by strong secondary, tertiary and quaternary interactions, including structures and interactions in complexes with proteins and RNAs. Potentially, comparisons between isoenergetic microarray results from *in vitro* transcribed RNA and RNA isolated from natural sources could be used to search for natural modified nucleotides within single-stranded regions of RNA. It should be particularly easy to detect modifications of functional groups involved in base pairing (such as N-alkyl, thio-modified nucleotides). Comparisons between microarray binding and secondary structures predicted by thermodynamics guided by chemical mapping can reveal unusual folding. For various reasons, an RNA may fold into multiple structures or its folding may be determined by kinetics rather than thermodynamics. In such cases, comparison of chemical mapping and microarray binding could discriminate between very different potential structures. For example, it has been proposed that encapsidated Satellite Tobacco Mosaic Virus (STMV) has a single structure (84) or an ensemble of structures (85,86). Very different binding to a microarray is predicted for each possibility.

Of course, there is a lot of room for improvements, including: (i) application of oligonucleotide probes containing 2-thiouridine residues to improve selectivity of base pairing with A in comparison to G (37,49,50), (ii) use of 3′-pyrene terminated oligonucleotide probes. The presence of pyrene enhances stability of LNA-2′MeRNA(probe)/RNA(target) duplexes by 2.3 ± 0.1 kcal/mol, independent of nucleotide in the opposite strand and the sequence of adjacent base pairs (56), (iii) development of computer programs for automatic analysis of hybridization results, including application of pseudo-ΔG^º^ restraints and evaluation of alternative binding sites and (iv) use of methods not requiring radioactivity to detect hybridization of target RNA to microarrays. These include fluorescence quenching (31), other label free methods (87) or mass spectrometry (88).

Applications of microarrays could be expanded to include probing more complex folding. For example: (i) tertiary interactions within RNA could be probed with spacer-linked oligonucleotides simultaneously complementary to different fragments of target RNA, and (ii) sequences allowing formation of bimolecular triplexes, quadruplexes and parallel duplexes could be identified.

## References

[1] Pirrung M.C., Southern E.M. (2014). The genesis of microarrays. Biochem. Mol. Biol. Educ..

[2] Maskos U., Southern E.M. (1992). Oligonucleotide hybridizations on glass supports—a novel linker for oligonucleotide synthesis and hybridization properties of oligonucleotides synthesized in situ. Nucleic Acids Res..

[3] Augenlicht L.H., Kobrin D. (1982). Cloning and screening of sequences expressed in a mouse colon-tumor. Cancer Res..

[4] Kulesh D.A., Clive D.R., Zarlenga D.S., Greene J.J. (1987). Identification of interferon-modulated proliferation-related cDNA sequences. Proc. Natl. Acad. Sci. U.S.A..

[5] Schena M., Shalon D., Davis R.W., Brown P.O. (1995). Quantitative monitoring of gene-expression patterns with a complementary-DNA microarray. Science.

[6] Southern E.M. (1975). Detection of specific sequences among DNA fragments separated by gel-electrophoresis. J. Mol. Biol..

[7] Fodor S.P.A., Read J.L., Pirrung M.C., Stryer L., Lu A.T., Solas D. (1991). Light-directed, spatially addressable parallel chemical synthesis. Science.

[8] Case-Green S.C., Mir K.U., Pritchard C.E., Southern E.M. (1998). Analysing genetic information with DNA arrays. Curr. Opin. Chem. Biol..

[9] Heller M.J. (2002). DNA microarray technology: devices, systems, and applications. Annu. Rev. Biomed. Eng..

[10] Ramsay G. (1998). DNA chips: state-of-the-art. Nat. Biotechnol..

[11] Hoheisel J.D. (2006). Microarray technology: beyond transcript profiling and genotype analysis. Nat. Rev. Genet..

[12] Merino E.J., Wilkinson K.A., Coughlan J.L., Weeks K.M. (2005). RNA structure analysis at single nucleotide resolution by selective 2′-hydroxyl acylation and primer extension (SHAPE). J. Am. Chem. Soc..

[13] Wilkinson K.A., Merino E.J., Weeks K.M. (2006). Selective 2′-hydroxyl acylation analyzed by primer extension (SHAPE): quantitative RNA structure analysis at single nucleotide resolution. Nat. Prot..

[14] Aviran S., Trapnell C., Lucks J.B., Mortimer S.A., Luo S., Schroth G.P., Doudna J.A., Arkin A.P., Pachter L. (2011). Modeling and automation of sequencing-based characterization of RNA structure. Proc. Natl. Acad. Sci. U.S.A..

[15] Lucks J.B., Mortimer S.A., Trapnell C., Luo S., Aviran S., Schroth G.P., Pachter L., Doudna J.A., Arkin A.P. (2011). Multiplexed RNA structure characterization with selective 2‘-hydroxyl acylation analyzed by primer extension sequencing (SHAPE-Seq). Proc. Natl. Acad. Sci. U.S.A..

[16] Kwok C.K., Ding Y., Tang Y., Assmann S.M., Bevilacqua P.C. (2013). Determination of *in vivo* RNA structure in low-abundance transcripts. Nat. Commun..

[17] Spitale R.C., Crisalli P., Flynn R.A., Torre E.A., Kool E.T., Chang H.Y. (2013). RNA SHAPE analysis in living cells. Nat. Chem. Biol..

[18] Sohail M., Akhtar S., Southern E.M. (1999). The folding of large RNAs studied by hybridization to arrays of complementary oligonucleotides. RNA.

[19] Sohail M., Hochegger H., Klotzbucher A., Le Guellec R., Hunt T., Southern E.M. (2001). Antisense oligonucleotides selected by hybridisation to scanning arrays are effective reagents in vivo. Nucleic Acids Res..

[20] Milner N., Mir K.U., Southern E.M. (1997). Selecting effective antisense reagents on combinatorial oligonucleotide arrays. Nat. Biotech..

[21] Mir K.U., Southern E.M. (1999). Determining the influence of structure on hybridization using oligonucleotide arrays. Nat. Biotech..

[22] Sohail M., Southern E.M. (2000). Hybridization of antisense reagents to RNA. Curr. Opin. Mol. Ther..

[23] Southern E.M. (1996). DNA chips: analysing sequence by hybridization to oligonucleotides on a large scale. Trends Genet..

[24] Southern E.M., Casegreen S.C., Elder J.K., Johnson M., Mir K.U., Wang L., Williams J.C. (1994). Arrays of complementary oligonucleotides for analyzing the hybridization behavior of nucleic acids. Nucleic Acids Res..

[25] Southern E.M., Maskos U., Elder J.K. (1992). Analyzing and comparing nucleic acid sequences by hybridization to arrays of oligonucleotides—evaluation using experimental models. Genomics.

[26] Southern E.M., Milner N., Mir K.U. (1997). Oligonucleotides as Therapeutic Agents.

[27] Maskos U., Southern E.M. (1992). Parallel analysis of oligodeoxyribonucleotide (oligonucleotide) interactions.1. Analysis of factors influencing oligonucleotide duplex formation. Nucleic Acids Res..

[28] Maskos U., Southern E.M. (1993). A novel method for the parallel analysis of multiple mutations in multiple samples. Nucleic Acids Res..

[29] Maskos U., Southern E.M. (1993). A study of oligonucleotide reassociation using large arrays of oligonucleotides synthesized on a glass support. Nucleic Acids Res..

[30] Sohail M., Doran G., Kang S., Akhtar S., Southern E.M. (2005). Structural rearrangements in RNA on the binding of an antisense oligonucleotide: implications for the study of intra-molecular RNA interactions and the design of cooperatively acting antisense reagents with enhanced efficacy. J. Drug Target..

[31] Duan S.H., Mathews D.H., Turner D.H. (2006). Interpreting oligonucleotide microarray data to determine RNA secondary structure: Application to the 3′ end of Bombyx mori R2 RNA. Biochemistry.

[32] Kierzek E. (2009). Binding of short oligonucleotides to RNA: studies of the binding of common RNA structural motifs to isoenergetic microarrays. Biochemistry.

[33] Kierzek E., Barciszewska M.Z., Barciszewski J. (2008). Isoenergetic microarray mapping reveals differences in structure between tRNA_i_^Met^ and tRNA_m_^Met^ from Lupinus luteus. Nucleic Acids Symp. Ser..

[34] Kierzek E., Christensen S.M., Eickbush T.H., Kierzek R., Turner D.H., Moss W.N. (2009). Secondary structures for 5′ regions of R2 retrotranspozon RNAs reveal a novel conserved pseudoknot and regions that evolve under different constraints. J. Mol. Biol..

[35] Kierzek E., Fratczak A., Pasternak A., Turner D.H., Kierzek R. (2007). Isoenergetic RNA microarrays, a new method to study the structure and interactions of RNA. Int. Proc. Division.

[36] Kierzek E., Kierzek R., Moss W.N., Christensen S.M., Eickbush T.H., Turner D.H. (2008). Isoenergetic penta- and hexanucleotide microarray probing and chemical mapping provide a secondary structure model for an RNA element orchestrating R2 retrotransposon protein function. Nucleic Acids Res..

[37] Kierzek E., Kierzek R., Turner D.H., Catrina I.E. (2006). Facilitating RNA structure prediction with microarrays. Biochemistry.

[38] Kierzek E., Mathews D.H., Ciesielska A., Turner D.H., Kierzek R. (2006). Nearest neighbor parameters for Watson-Crick complementary heteroduplexes formed between 2′-O-methyl RNA and RNA oligonucleotides. Nucleic Acids Res..

[39] Xia T.B., SantaLucia J., Burkard M.E., Kierzek R., Schroeder S.J., Jiao X.Q., Cox C., Turner D.H. (1998). Thermodynamic parameters for an expanded nearest-neighbor model for formation of RNA duplexes with Watson-Crick base pairs. Biochemistry.

[40] Sugimoto N., Nakano S., Katoh M., Matsumura A., Nakamuta H., Ohmichi T., Yoneyama M., Sasaki M. (1995). Thermodynamic parameters to predict stability of RNA/DNA hybrid duplexes. Biochemistry.

[41] Zuker M. (2003). Mfold web server for nucleic acid folding and hybridization prediction. Nucleic Acids Res..

[42] Reuter J.S., Mathews D.H. (2010). RNAstructure: software for RNA secondary structure prediction and analysis. BMC Bioinformatics.

[43] Serra M.J., Baird J.D., Dale T., Fey B.L., Retatagos K., Westhof E. (2002). Effects of magnesium ions on the stabilization of RNA oligomers of defined structures. RNA.

[44] Ciesiolka J., Lorenz S., Erdmann V.A. (1992). Different conformational forms of Escherichia coli and rat-liver 5S ribosomal-RNA revealed by Pb(II) induced hydrolysis. Eur. J. Biochem..

[45] Nazar R.N. (1991). Higher-order structure of the ribosomal 5S RNA. J. Biol. Chem..

[46] Freier S.M., Kierzek R., Caruthers M.H., Neilson T., Turner D.H. (1986). Free-energy contributions of G-U and other terminal mismatches to helix stability. Biochemistry.

[47] Chen J.L., Dishler A.L., Kennedy S.D., Yildirim I., Liu B., Turner D.H., Serra M.J. (2012). Testing the nearest neighbor model for canonical RNA base pairs: revision of GU parameters. Biochemistry.

[48] Sugimoto N., Kierzek R., Freier S.M., Turner D.H. (1986). Energetics of internal GU mismatches in ribooligonucleotide helixes. Biochemistry.

[49] Testa S.M., Disney M.D., Turner D.H., Kierzek R. (1999). Thermodynamics of RNA-RNA duplexes with 2-or 4-thiouridines: Implications for antisense design and targeting a group I intron. Biochemistry.

[50] Carlucci M., Kierzek E., Olejnik A., Turner D.H., Kierzek R. (2009). Chemical synthesis of LNA-2-thiouridine and its influence on stability and selectivity of oligonucleotide binding to RNA. Biochemistry.

[51] Kierzek E., Ciesielska A., Pasternak K., Mathews D.H., Turner D.H., Kierzek R. (2005). The influence of locked nucleic acid residues on the thermodynamic properties of 2′-O-methyl RNA/RNA heteroduplexes. Nucleic Acids Res..

[52] Koshkin A.A., Singh S.K., Nielsen P., Rajwanshi V.K., Kumar R., Meldgaard M., Olsen C.E., Wengel J. (1998). LNA (Locked Nucleic Acids): Synthesis of the adenine, cytosine, guanine, 5-methylcytosine, thymine and uracil bicyclonucleoside monomers, oligomerisation, and unprecedented nucleic acid recognition. Tetrahedron.

[53] Obika S., Nanbu D., Hari Y., Morio K., In Y., Ishida T., Imanishi T. (1997). Synthesis of 2′-O,4′-C-methyleneuridine and -cytidine. Novel bicyclic nucleosides having a fixed C-3′-endo sugar puckering. Tetrahedron Lett..

[54] Wengel J., Petersen M., Frieden M., Koch T. (2003). Chemistry of locked nucleic acids (LNA): Design, synthesis, and biophysical properties. Lett. Pept. Sci..

[55] Pasternak A., Kierzek E., Pasternak K., Turner D.H., Kierzek R. (2007). A chemical synthesis of LNA-2,6-diaminopurine riboside, and the influence of 2′-O-methyl-2,6-diaminopurine and LNA-2,6-diaminopurine ribosides on the thermodynamic properties of 2′-O-methyl RNA/RNA heteroduplexes. Nucleic Acids Res..

[56] Pasternak A., Kierzek E., Pasternak K., Fratczak A., Turner D.H., Kierzek R. (2008). The thermodynamics of 3′-terminal pyrene and guanosine for the design of isoenergetic 2′-O-methyl-RNA-LNA chimeric oligonucleotide probes of RNA structure. Biochemistry.

[57] Timofeev E.N., Kochetkova S.V., Mirzabekov A.D., Florentiev V.L. (1996). Regioselective immobilization of short oligonucleotides to acrylic copolymer gels. Nucleic Acids Res..

[58] Timofeev E., Mirzabekov A. (2001). Binding specificity and stability of duplexes formed by modified oligonucleotides with a 4096-hexanucleotide microarray. Nucleic Acids Res..

[59] Hart J.M., Kennedy S.D., Mathews D.H., Turner D.H. (2008). NMR-assisted prediction of RNA secondary structure: Identification of a probable pseudoknot in the coding region of an R2 Retrotransposon. J. Am. Chem. Soc..

[60] Clanton-Arrowood K., McGurk J., Schroeder S.J. (2008). 3′ terminal nucleotides determine thermodynamic stabilities of mismatches at the ends of RNA helices. Biochemistry.

[61] Ohmichi T., Nakano S., Miyoshi D., Sugimoto N. (2002). Long RNA dangling end has large energetic contribution to duplex stability. J. Am. Chem. Soc..

[62] Liang R.T., Kierzek E., Kierzek R., Turner D.H. (2010). Comparisons between chemical mapping and binding to isoenergetic oligonucleotide microarrays reveal unexpected patterns of binding to the Bacillus subtilis RNase P RNA specificity domain. Biochemistry.

[63] Jenek M., Kierzek E. (2008). Isoenergetic microarray mapping—the advantage of this method in studying the structure of Saccharomyces cerevisiae tRNA^Phe^. Nucleic Acids Symp. Ser..

[64] Krasilnikov A.S., Yang X.J., Pan T., Mondragon A. (2003). Crystal structure of the specificity domain of ribonuclease P. Nature.

[65] Berkhout B., Ooms M., Beerens N., Huthoff H., Southern E., Verhoef K. (2002). In vitro evidence that the untranslated leader of the HIV-1 genome is an RNA checkpoint that regulates multiple functions through conformational changes. J. Biol. Chem..

[66] Jiang T., Kennedy S.D., Moss W.N., Kierzek E., Turner D.H. (2014). Secondary structure of a conserved domain in an intron of influenza A M1 mRNA. Biochemistry.

[67] Moss W.N., Dela-Moss L.I., Kierzek E., Kierzek R., Priore S.F., Turner D.H. (2012). The 3′ splice site of influenza a segment 7 mRNA can exist in two conformations: a pseudoknot and a hairpin. Plos One.

[68] Moss W.N., Dela-Moss L.I., Priore S.F., Turner D.H. (2012). The influenza A segment 7 mRNA 3′ splice site pseudoknot/hairpin family. RNA Biol..

[69] Priore S.F., Kierzek E., Kierzek R., Baman J.R., Moss W.N., Dela-Moss L.I., Turner D.H. (2013). Secondary structure of a conserved domain in the intron of influenza A NS1 mRNA. Plos One.

[70] Huthoff H., Berkhout B. (2002). Multiple secondary structure rearrangements during HIV-1 RNA dimerization. Biochemistry.

[71] Moss W.N., Priore S.F., Turner D.H. (2011). Identification of potential conserved RNA secondary structure throughout influenza A coding regions. RNA.

[72] Sipa K., Sochacka E., Kazmierczak-Baranska J., Maszewska M., Janicka M., Nowak G., Nawrot B. (2007). Effect of base modifications on structure, thermodynamic stability, and gene silencing activity of short interfering RNA. RNA.

[73] Rife J.P., Cheng C.S., Moore P.B., Strobel S.A. (1998). N-2-methylguanosine is iso-energetic with guanosine in RNA duplexes and GNRA tetraloops. Nucleic Acids Res..

[74] Byrne R.T., Konevega A.L., Rodnina M.V., Antson A.A. (2010). The crystal structure of unmodified tRNA(Phe) from Escherichia coli. Nucleic Acids Res..

[75] Michalowski D., Wrzesinski J., Ciesiolka J., Krzyzosiak W.J. (1996). Effect of modified nucleotides on structure of yeast tRNA(Phe). Comparative studies by metal ion-induced hydrolysis and nuclease mapping. Biochimie.

[76] Sampson J.R., Uhlenbeck O.C. (1988). Biochemical and physical characterization of an unmodified yeast phenylalanine transfer-RNA transcribed in vitro. Proc. Natl. Acad. Sci. U.S.A..

[77] Fratczak A., Kierzek R., Kierzek E. (2011). Isoenergetic microarrays to study the structure and interactions of DsrA and OxyS RNAs in two- and three-component complexes. Biochemistry.

[78] Lease R.A., Belfort M. (2000). A trans-acting RNA as a control switch in Escherichia coli: DsrA modulates function by forming alternative structures. Proc. Natl. Acad. Sci. U.S.A..

[79] Rolle K., Zywicki M., Wyszko E., Barciszewska M.Z., Barciszewski J. (2006). Evaluation of the dynamic structure of DsrA RNA from E.coli and its functional consequences. J. Biochem..

[80] Lease R.A., Woodson S.A. (2004). Cycling of the Sm-like protein Hfq on the DsrA small regulatory RNA. J. Mol. Biol..

[81] Ahlborn C., Siegmund K., Richert C. (2007). Isostable DNA. J. Am. Chem. Soc..

[82] SantaLucia J., Allawi H.T., Seneviratne A. (1996). Improved nearest-neighbor parameters for predicting DNA duplex stability. Biochemistry.

[83] Gude L., Berkovitch S.S., Santos W.L., Kutchukian P.S., Pawloski A.R., Kuimelis R., McGall G., Verdine G.L. (2012). Mapping targetable sites on human telomerase RNA pseudoknot/template domain using 2′-OMe RNA-interacting polynucleotide (RIPtide) microarrays. J. Biol. Chem..

[84] Archer E.J., Simpson M.A., Watts N.J., O'Kane R., Wang B., Erie D.A., McPherson A., Weeks K.M. (2013). Long-range architecture in a viral RNA genome. Biochemistry.

[85] Schroeder S.J., Stone J.W., Bleckley S., Gibbons T., Mathews D.M. (2011). Ensemble of secondary structures for encapsidated satellite Tobacco Mosaic Virus RNA consistent with chemical probing and crystallography constraints. Biophys. J..

[86] Schroeder S.J. (2014). Probing viral genomic structure: alternative viewpoints and alternative structures for Satellite Tabacco Mosaic Virus RNA. Biochemistry.

[87] Pan S.L., Rothberg L.J. (2003). Interferometric sensing of biomolecular binding using nanoporous aluminum oxide templates. Nano Lett..

[88] Zhang X.Y., Zhu S.C., Deng C.H., Zhang X.M. (2012). An aptamer based on-plate microarray for high-throughput insulin detection by MALDI-TOF MS. Chem. Commun..

